# Broad-scale predictions of herpetofauna occupancy and colonization in an agriculturally dominated landscape

**DOI:** 10.1371/journal.pone.0306655

**Published:** 2024-10-30

**Authors:** Jonathan P. Harris, Tyler M. Harms, Karen E. Kinkead, Paul W. Frese, David M. Delaney, Emma M. Buckardt, Stephen J. Dinsmore

**Affiliations:** 1 Department of Natural Resource Ecology and Management, Iowa State University, Ames, Iowa, United States of America; 2 Center for Survey Statistics and Methodology, Iowa State University, Ames, Iowa, United States of America; 3 Iowa Department of Natural Resources, Boone, Iowa, United States of America; Instituto Federal de Educacao Ciencia e Tecnologia Goiano - Campus Urutai, BRAZIL

## Abstract

Predictions of species occurrence allow land managers to focus conservation efforts on locations where species are most likely to occur. Such analyses are rare for herpetofauna compared to other taxa, despite increasing evidence that herptile populations are declining because of landcover change and habitat fragmentation. Our objective was to create predictions of occupancy and colonization probabilities for 15 herptiles of greatest conservation need in Iowa. From 2006–2014, we surveyed 295 properties throughout Iowa for herptile presence using timed visual-encounter surveys, coverboards, and aquatic traps. Data were analyzed using robust design occupancy modeling with landscape-level covariates. Occupancy ranged from 0.01 (95% CI = -0.01, 0.03) for prairie ringneck snake (*Diadophis punctatus arnyi*) to 0.90 (95% CI = 0.898, 0.904) for northern leopard frog (*Lithobates pipiens*). Occupancy for most species correlated to landscape features at the 1-km scale. General patterns of species’ occupancy included negative effects of agricultural features and positive effects of water features on turtles and frogs. Colonization probabilities ranged from 0.007 (95% CI = 0.006, 0.008) for spiny softshell turtle (*Apalone spinifera*) to 0.82 (95% CI = 0.62, 1.0) for western fox snake (*Pantherophis ramspotti*). Colonization probabilities for most species were best explained by effects of water and grassland landscape features. Predictive models had strong support (AUC > 0.70) for six out of 15 species (40%), including all three turtles studied. Our results provide estimates of occupancy and colonization probabilities and spatial predictions of occurrence for herptiles of greatest conservation need across the state of Iowa.

## Introduction

Conserving wildlife populations hinges on the knowledge of species’ habitat requirements. Common methods for evaluating species habitat requirements, such as logistic regression and occupancy modeling [1], rely on presence-absence data for a collection of sampled sites to estimate the probability of occurrence based on biotic (e.g., local habitat characteristics) and abiotic (e.g., environmental variables) factors. These methods are popular because presence-absence data are logistically feasible and cheaper to collect relative to other data types [2], such as movement data or mark-recapture, thereby allowing additional sample collections across a larger geographic area. Evaluating species habitat requirements at a collection of sampled sites can be particularly effective at informing habitat restoration and management, but inference can be limited to sampled locations and scaling up to broader predictions on species’ distributions can be challenging [3] depending on geographic scope and property selection design. Predictive models that incorporate species’ presence-absence data and environmental data over large spatial extents can provide broader inferences of ecological processes and species’ distributions, thereby aiding the prioritization of areas for conservation, habitat restoration, and management efforts [4, 5].

Local habitat characteristics are critical for the success of each life stage of a species [6] and can affect breeding success, the accessibility of shelter during inclement weather, and food availability [7]. However, landscape-level habitat characteristics, such as connectivity and other patch dynamics, can also affect the distribution of a species and influence metapopulation dynamics [8, 9]. In other words, a single patch may contain local habitat characteristics for reproductive success, but may be isolated on the landscape, preventing initial colonization or dispersal after breeding [10]. Human modifications to landscapes are continuously increasing, primarily due to agriculture and urban development, resulting in shifting wildlife distributions [11]. These broad-scale changes in landcover and connectivity are likely significant contributors to biodiversity loss [12–14]. In Iowa, USA, the majority of the landscape has been modified for agriculture, resulting in less than one percent of remnant prairies and five percent of remnant wetlands [15, 16], correlating with statewide losses in biodiversity [17]. Currently, the Iowa landscape contains approximately 2.1% wetlands/open water and 21.2% grasslands and pastures [16].

Herpetofauna (amphibians and reptiles) are at a greater risk of extinction than birds or mammals [18], but have been understudied relative to other terrestrial vertebrates [19, 20]. Increasing evidence suggests that the greatest threats to herptile populations are likely agricultural development and other landscape alterations, such as habitat fragmentation [21–23], particularly in the northern-most regions of species’ ranges [23]. Pesticide application in agricultural landscapes can have direct effects on amphibian populations through mortality [24] and potentially indirect effects on habitat use [25], skewed sex ratios [26], and predator-prey interactions, where pesticide exposure has been shown to increase predation risk and mortality in tadpoles [27, 28]. Habitat fragmentation may be more important to herptiles relative to other taxa, given their limited dispersal abilities [29, 30]. Numerous studies have evaluated influences of landscape characteristics on home range size [30–32], movement dynamics [33], habitat selection [30, 34–36], and relative abundance [37] of reptiles and amphibians. Although studies on herpetofauna patch occupancy are becoming more common [38–43], few studies have used occupancy modeling to predict herpetofauna occupancy and colonization over broad spatial scales [40, 44–46].

The ability to predict patterns of species occurrence based on landscape characteristics aids in conservation planning because it allows for allocation of limited resources to habitat management and restoration in areas that will most benefit species of conservation concern. Additionally, with the emergence of robust-design occupancy modeling [47], insights can be made into metapopulation metrics, such as colonization probabilities. Spatial predictions of colonization probabilities may be a useful conservation tool to target locations where increasing connectivity can be a habitat management priority [48, 49], particularly for species with limited dispersal capabilities.

We sought to understand how agricultural development and landcover characteristics affect herptile presence in Iowa. Specifically, our objective was to create spatially predictive models of herptile occupancy and colonization probabilities to help inform conservation planning for species of greatest conservation need (SGCN). Iowa’s Department of Natural Resources designates species with low and declining populations as SGCN in the Iowa Wildlife Action Plan [16]. There are 68 species (22 amphibians, 46 reptiles) of herptiles in Iowa, with 56 species considered SGCN under the 2015 Iowa Wildlife Action Plan [16]. We focused this analysis on 15 of the 56 SGCN species (Table 1). Species were included in analysis if they were SGCN and if there were enough detections for model convergence.

**Table 1:** Herptile species with the proportion of sites with detections across all years (naive occupancy, $\tilde{\psi }$) and modeled estimates for initial occupancy (*ψ*), colonization (γ), and detection (p) probabilities in Iowa, 2006–2014. Modeled estimates were derived from the top model for each species and include 95% confidence intervals. We did not estimate local extinction probabilities because we did not have sufficient data for some species for the full model to converge and we wanted a numerical estimate of occupancy. Species are organized by amphibians in alphabetical order, followed by reptiles in alphabetical order.

Species	$\tilde{\psi }$	*ψ*	95% CI	γ	95% CI	p	95% CI
Blanchard’s Cricket Frog (*Acris blanchardi*)	0.74	0.76	(0.70, 0.82)	0.21	(0.05, 0.37)	0.73	(0.74, 0.76)
Gray Treefrog Complex (*Hyla spp*)	0.38	0.44	(0.38, 0.50)	0.36	(0.34,0.37)	0.03	(0.01, 0.08)
Northern Leopard Frog (*Lithobates pipiens*)	0.90	0.90	(0.89, 0.90)	0.44	(0.36, 0.52)	0.64	(0.60, 0.68)
Southern Leopard Frog (*Lithobates sphenocephalus*)	0.16	0.09	(0.03, 0.15)	NE	NE	0.29	(0.19, 0.39)
Tiger Salamander (*Ambystoma tigrinum*)	0.06	0.04	(0.03, 0.05)	0.02	(0.016, 0.024)	0.01	(-0.01, 0.03)
Blanding’s Turtle (*Emydoidea blandingii*)	0.04	0.06	(0.04, 0.08)	NE	NE	0.65	(0.10, 1.2)
Common Snapping Turtle (*Chelydra serpentina*)	0.43	0.58	(0.48, 0.68)	0.22	(-0.02, 0.46)	0.27	(0.23, 0.31)
Northern Redbelly Snake (*Storeria occipitomaculata*)	0.2	0.11	(0.108, 0.113)	0.08	(0.076, 0.084)	0.28	(0.20, 0.36)
Northern Watersnake (*Nerodia sipedon*)	0.29	0.34	(0.32, 0.36)	0.03	(0.02, 0.04)	0.47	(0.39, 0.55)
Plains Garter Snake (*Thamnophis radix*)	0.31	0.43	(0.33, 0.53)	0.19	(-0.03, 0.41)	0.24	(0.20, 0.28)
Prairie Ringneck Snake (*Diadophis punctatus arnyi*)	0.11	0.01	(-0.01, 0.03)	NE	NE	0.26	(-0.11, 0.63)
Prairie Skink (*Plestiodon septentrionalis*)	0.16	0.18	(0.12, 0.24)	NE	NE	0.38	(0.28, 0.48)
Spiny Softshell Turtle (*Apalone spinifera*)	0.15	0.19	(0.17, 0.21)	0.01	(0.008, 0.012)	0.18	(0.12, 0.24)
Western Fox Snake (*Pantherophis ramspotti*)	0.11	0.09	(0.05, 0.13)	0.82	(0.62, 1.02)	0.03	(-0.03, 0.09)
Western Ribbon Snake (*Thamnophis proximus*)	0.16	0.21	(0.09, 0.33)	NE	NE	0.17	(0.11, 0.23)

NE, Not Estimated.

## Methods

### Study area and site selection

Iowa’s landscape is dominated by agriculture consisting of seven distinct landform regions: Northwest Iowa Plains, Missouri Alluvial Plain, Western Loess Hills, Des Moines Lobe, Southern Iowa Drift Plain, Iowan Surface, Paleozoic Plateau, and Mississippi Alluvial Plain [49]. Historically, these landform regions produced a variety of habitats including tallgrass prairie, savannas, and pothole wetlands that comprised most of the Iowa landscape. Although some of these habitats remain, 63% of Iowa is now agricultural land and 2.5% is developed [50].

**Fig 1:**
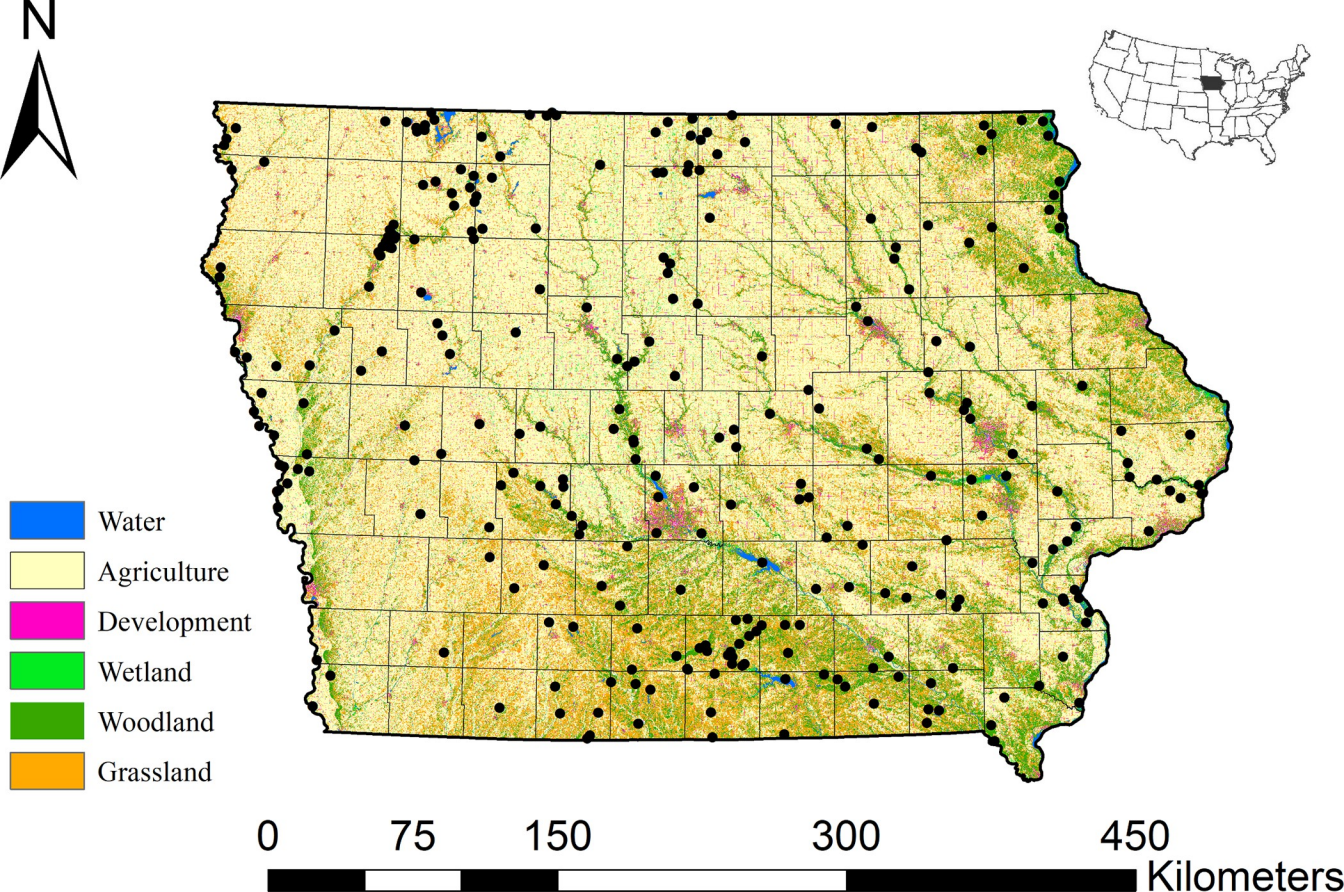
Sites surveyed for herptiles (black dots) in Iowa, 2006–2014. Basemap data provided by [65].

As part of the Iowa Multiple Species Inventory and Monitoring (MSIM) program [51] we conducted our study on 295 public properties greater than 97 ha (Fig 1, S1 Table). A small number of private properties were included in our study to document SGCN on lands enrolled in the Landowner Incentive Program (see http://wsfrprograms.fws.gov/subpages/grantprograms/lip/lip.htm). We classified all properties according to 19 different habitat types (S1 Table) listed in the 2005 Iowa Wildlife Action Plan [50] using aerial imagery, 2002 Iowa landcover data in ArcGIS (ver. 10.1) [52], and knowledge of the local land manager. We classified properties by one or more habitat types depending on the degree of habitat diversity on the property. We then divided Iowa into four equal management districts for property selection to allow for equal representation of all habitat types across the state. We utilized a stratified random sampling technique to select properties which involved selecting properties of a specified habitat type (primary stratum) within each management district (secondary stratum). Once a property was selected for a particular habitat type, it was excluded from selection for a different habitat type. We then defined the “core” habitat type as the habitat type for which the property was selected. We repeated this procedure from 2006 to 2014 to obtain approximately 75 properties to survey each year. Beginning in 2017, we selected at least two properties each year to be surveyed annually. In this way, we increased the number of annual properties each year, resulting in 27 properties surveyed for three or more years, 18 surveyed for four or more years, and 10 surveyed for five or more years (S1 Table). The procedure for selecting annual properties was similar to that mentioned above, although we limited our selection to those properties owned and managed by the Iowa Department of Natural Resources Wildlife Bureau for ease of logistics. We obtained Special Use Permits from the U.S. Fish and Wildlife Service for data collection on National Wildlife Refuges (Permit # 2013–016), and a Scientific Collector’s Permit from the Iowa Department of Natural Resources (Permit # SC872). All field methods for this study were reviewed and approved by the Iowa State University Institutional Animal Care and Use Committee (IACUC; Protocol #3-12-7326-Z).

### Herptile surveys

We used three different methods for surveying herptiles. Occurrence data collected using all methods were compiled for all years to establish a single occurrence history for each species on each property. Properties were also clipped to the known ranges of each species to increase accuracy for predictive models, resulting in unequal sample sizes for each species.

We conducted standardized visual encounter surveys for herptiles each year from April to October in the period 2006–2014. This survey method involved a timed search for herptiles in suitable habitats identified and mapped on each property at the beginning of the survey year. We divided each year into three survey seasons to minimize seasonal variation in detection probability of different species. Those seasons were spring (15 April–15 June), summer (16 June–15 August), and fall (16 August–15 October). We surveyed each property for four person-hours twice during each of the three survey seasons for a maximum of six visits (24 person-hours) per year. We surveyed properties only during the year in which they were selected except for those properties that were selected for annual surveys. Surveys were conducted two weeks apart on average to increase independence among visits. During the spring and fall, we conducted surveys during warmer hours of the day, which typically fell between 10:00 hours and 18:00 hours, to maximize detection probabilities. We did not perform nocturnal vocalization surveys for technician safety purposes. Two species, Cope’s gray treefrog (*Hyla chrysoscelis*) and eastern gray treefrog (*Hyla versicolor*), can only be distinguished via auditory detections. Given that we could not confidently differentiate between the two species, we combined their detection histories and modeled both species in aggregate as the gray treefrog complex.

We also placed coverboards at both systematic and random locations on the property to increase detection probabilities for herptiles. Six coverboards were placed systematically in a hexagonal arrangement 200 m apart in the core habitat type on the property and an additional nine coverboards were placed in locations that seemed most likely to capture individuals, for a total of 15 coverboards per property. Coverboards are frequently used by many species of herpetofauna as habitats for thermoregulation and cover, but may be a more efficient method for capturing reptiles than amphibians [53–56]. Each coverboard was marked with a global positioning system (GPS) unit and checked during each visual encounter survey. All herptiles observed during the survey were identified to species [57]. Surveys were not conducted on cool days (< 10°C) or during periods of rain.

Lastly, we used aquatic traps to target amphibians and turtles. We set a variety of aquatic traps, which typically consisted of three hoop nets, three box traps, three fish traps, and up to six minnow traps, for two trap nights once per each of three survey seasons described above for a total of six trap nights per property. We identified water bodies on each property, which included ponds, wetlands, lakes, rivers, and streams. Aquatic traps were placed within waterbodies at locations deemed most likely for captures. We baited larger traps with fish (e.g., sardines, dead grass carp [*Ctenopharyngodon idella*]) and checked traps once daily. We replaced bait as needed when traps were checked.

### Habitat variables

We used ArcGIS (ver. 10.1) [46] to measure landscape-level habitat variables within 200-m, 500-m, and 1000-m radii of each property. Buffers at each scale were placed around each survey mid-point for each property using the buffer tool in ArcGIS toolbox (Analysis Tools, Proximity, Buffer) [52]. We then clipped a 2009 Iowa Landcover file, developed by the Iowa Department of Natural Resources, to all buffers at all properties using the “clipraster” command in the Geospatial Modeling Environment (GME) package [58]. The 2009 Iowa Landcover was developed using satellite imagery and includes 15 classifications such as coniferous and deciduous forests, corn and soybean crops, and anthropogenic structures, among others, at a 3-m resolution. We then aggregated the classifications into six landcover types: water, agriculture, development, wetland, woodland, and grassland. These landcover types were selected due to their potential influence on our focal species and their interest to land managers. We opted to use landcover data for Iowa from 2009 because it is centered in our time series of surveys (2006–2014) and because the file is higher resolution (3m) than most other landcover products.

We used FRAGSTATS (ver. 3.4) [59] to summarize landcover metrics at each buffer. FRAGSTATS is a computer program that analyzes spatial pattern based on categorical maps and allows the user to pick from a variety of metrics to assess landscape configuration [59]. We used the percentage of landscape (PLND), largest patch index (LPI), patch density (PD), and edge density (ED) for all analyses. Percentage of landscape is the area of a land-use classification divided by the total area of the landscape. Largest patch index is the area of the largest patch of a land-use classification divided by the total area of the landscape. Patch density is the count of patches corresponding to a land-use classification divided by the landscape area. Edge density is the amount of linear edge on the landscape for a land-use classification divided by landscape area. We extracted each FRAGSTATS metric for each of the six landcover types within three different spatial scales (200, 500, and 1000-m radii) to be included as prediction covariates in our models.

### Predictive models

We used a robust design occupancy model framework [47] in Program Mark [60] to estimate occupancy and colonization probabilities for 15 herptile species in Iowa (Table 1). Robust design occupancy models account for imperfect detection across multiple survey years [41], allowing for the estimation of probability of initial occupancy (ψ), probability of local colonization (γ), probability of local extinction (ε), and detection probability (p) [47] as response variables. Unlike the single-season occupancy model where sites are closed to changes in occupancy state during the primary sampling season [1], the robust design occupancy model assumes sites are closed to changes in occupancy state between secondary sampling intervals (e.g., sampling occasions within a year) but are open to changes in occupancy state between primary sampling intervals (e.g., years) [47]. This allows for the evaluation of metapopulation dynamics through the process of determining the probability a site will remain occupied or become colonized. We used real parameter estimates for occupancy, which represent the initial occupancy probability at the first year of survey. The first year of survey varies by property, as properties were continually added throughout the survey period. We did not estimate local extinction probabilities for two reasons. First, we did not have sufficient data for some species for the full model to converge, so we chose to focus inferences on occupancy and colonization. Second, we wanted a numerical estimate of occupancy as opposed to one derived recursively from colonization and extinction probabilities [47]. One possible source of bias is that these models do not account for spatial autocorrelation. However, all properties were separated by a minimum distance of 530 m and were on average 10.2 km away from the nearest property, which we deemed adequate to meet the assumption of spatial independence.

Landscape-level habitat variables (S2 Table, S3 Table) and detection covariates were included in occupancy model sets as predictor variables along with intercept-only null models. The primary sampling intervals were the survey years (2006–2012, 2014) and the secondary sampling intervals were the survey occasions (days) within each sampling year (April-October). Data from 2013 were excluded from model building and later used for testing the accuracy of predictive models. We chose 2013 for model testing because it was one of the final years of surveys and more properties were surveyed in 2013 (n = 74) than in 2014 (n = 27). We estimated probability of occupancy and colonization on primary sampling intervals, and detection probability on secondary sampling intervals. Models were constructed in a hierarchical fashion separately for each species [61] and evaluated using Akaike’s Information Criterion adjusted for small sample sizes (AICc) [62]. Models with ΔAICc ≤ 2 were considered to have strong support [62]. We first modeled environmental covariates (e.g., temperature [˚F], percent cloud cover) on detection probability while keeping all other parameters constant. Using the best model from this step, we then modeled landscape-level covariates on probability of occupancy. Our goal was to identify the most important landscape-level factor that predicts occupancy and colonization probabilities for each species. Therefore, we compared each predictor in separate models to identify the variable with the most explanatory power, except for two species (northern redbelly snake and gray tree frog complex) where life-history characteristics were a priori expected to depend on environmental interactions (as determined by LeClere [57], S4 Table). We then selected the best model with effects on detection probability and probability of occupancy to be used in modeling effects on probability of colonization. Among the top models, we compared model deviance as an indicator of goodness-of-fit. We ultimately determined the top ranked model to be the best for predictions for every species, given that model averaging does not increase prediction accuracy, even when the top models have similar AICc weights [63]. We used the top model as a basis for inference to build our predictive maps.

To predict probability of occupancy across Iowa for each species, we first established a 1000-m point grid across the entire state using the tools extension package NPS AlaskaPak [64]. We used these points to extract landscape-level habitat characteristics of interest across Iowa. We used the same process described above for our sampled properties to estimate landscape-level habitat characteristics for each of the four land-use classifications, where the 2009 Iowa Landcover file was clipped to three buffers (200 m, 500 m, and 1000 m radius) around each point in the grid. We then developed predictive models for each parameter for each species using the linear coefficients from the top occupancy model. We calculated a probability of occupancy and colonization for each point in the point grid by taking the logit transformation of the product of the linear coefficient of the covariate in the top model (corresponding to the parameter of interest) and the value for the covariate at the respective point. The probabilities for all points in the grid were mapped using the raster conversion tool (Conversion Tools, To Raster, Point to Raster) [52] to create a raster corresponding to the predicted probability.

Using data from 2013, we tested the validity of our models using the area under the receiver operating characteristic curve (AUC). An AUC score of 0.5 suggests that the model prediction did not successfully separate true predictions from false predictions, compared to a score of 1.0, where all occurrence points were predicted correctly [65]. We considered models useful if AUC was ≥ 0.70 [65].

Lastly, we combined the predictive maps for all species using the Raster Calculator tool to sum the predictive occupancy rasters for all species with an AUC ≥ 0.70. The resulting raster provides a SGCN herptile hotspot map for Iowa.

## Results

We found 55 species of herptiles, including 45 SGCN, from 2006–2014 across all sites. Northern leopard frog (*Lithobates pipiens*) was the most commonly found SGCN species, with at least one detection at 90% of properties (Table 1). Several species were only observed once, including eastern massasauga (*Sistrurus catenatus*), blue-spotted salamander (*Ambystoma laterale*), plains spadefoot toad (*Spea bombifrons*), and western worm snake (*Carphophis vermis*). Tiger salamander (*Ambystoma tigrinum*) was the rarest species included in our models, found at 6% of properties (Table 1).

Most species responded to landscape features at the 1000-m scale for site occupancy, including all frog species (Table 2). Conversely, the best scale for predicting colonization probabilities was more varied across species. Tiger salamander was the only species that was best predicted at the 200-m scale for both occupancy and colonization.

**Table 2:** The best models for herptile occupancy (*ψ*), colonization (γ), and detection probabilities (p), as a function of landscape characteristics and detection covariates in Iowa, 2006–2014. The value and 95% CI represent the linear coefficients for the covariate modeled on the respective parameter.

Species	Model		95% CI	γ	95% CI	p	95% CI
Blanchard’s Cricket Frog (*Acris blanchardi*)	Ψ (~Ag1KPLND) γ (~Grs500PD) p(~Wind)	-0.03	(-0.05, -0.02)	0.01	(0.000,0.01)	-0.03	(-0.06, -0.01)
Gray Treefrog Complex (*Hyla spp*)	Ψ (~Wod1KPLND) γ (~Wtl500PLND) p(~Temp)	0.04	(0.03, 0.05)	-0.25	(-0.27, -0.24)	0.03	(0.02, 0.05)
Northern Leopard Frog (*Lithobates pipiens*)	Ψ (~Ag1KPD) γ (~Wtl500LPI) p(~Wind)	-0.003	(-0.003,-0.003)	-0.84	(-1.13, -0.56)	0.06	(0.03, 0.08)
Southern Leopard Frog (*Lithobates sphenocephalus*)	Ψ (~Wtr1KPD) γ (~Grs200LPI) p(~1)	0.02	(0.01, 0.02)	-0.59	(-1.90, 0.72)		
Tiger Salamander (*Ambystoma tigrinum*)	Ψ (~Ag200ED) γ (~Wtl200PD) p(~Temp)	0.003	(0.002, 0.003)	0.01	(0.01, 0.01)	0.06	(0.02, 0.11)
Blanding’s Turtle (*Emydoidea blandingii*)	Ψ (~Wtr200ED) γ (~Grs1KPLND) p(~Temp)	0.01	(0.01, 0.01)	-7.2	(-7.3, -7.09)	-0.04	(-0.07, -0.01)
Common Snapping Turtle (*Chelydra serpentina*)	Ψ (~Wod1KPD) γ (~Ag500LPI) p(~1)	-0.004	(-0.01, 0.0004)	-0.2	(-0.42, 0.01)		
Spiny Softshell Turtle (*Apalone spinifera*)	Ψ (~Ag200PD) γ (~Wtr1KPD) p(~1)	-0.002	(-0.002, -0.002)	0.02	(0.02, 0.02)		
Northern Redbelly Snake (*Storeria occipitomaculata*)	Ψ (~Ag500PLND) γ (~Wod1kED * Wtl1kED) p(~Wind)	-0.24	(-0.25, -0.24)	-0.02	(-0.02, -0.02)	0.08	(0.03, 0.14)
Northern Watersnake (*Nerodia sipedon*)	Ψ (~Ag1KPLND) γ (~Ag500PD) p(~Cld)	-0.07	(1.07, 1.19)	-0.01	(-0.01, -0.01)	-0.01	(-0.01, -0.002)
Plains Garter Snake (*Thamnophis radix*)	Ψ (~Wtr500LPI) γ (~Wtl1KPD) p(~Wind)	-0.09	(-0.16, -0.01)	-0.02	(-0.04, 0.004)	0.2	(-0.01, 0.41)
Prairie Ringneck Snake (*Diadophis punctatus arnyi*)	Ψ (~Ag200LPI) γ (~Wtr1KPLND) p(~Cld)	-0.44	(-0.77, -0.10)	-3.16	(-6.40, 0.09)	0.01	(0.001, 0.02)
Prairie Skink (*Plestiodon septentrionalis*)	Ψ (~Grs1kLPI) γ (~Grs1KPLND) p(~Cld)	0.08	(0.04, 0.12)	1.08	(-0.53, 2.70)	0.01	(-0.001, 0.02)
Western Fox Snake (*Pantherophis ramspotti*)	Ψ (~Wtr1KPLND) γ (~Wtr1kLPI) p(~Wind)	0.04	(0.03, 0.05)	0.88	(0.68, 1.08)	0.02	(-0.01, 0.04)
Western Ribbon Snake (*Thamnophis proximus*)	Ψ (~Wtl1kLPI) γ (~Grs200PLND) p(~1)	0.38	(0.01, 0.75)	12.23	(-81.5, 105.95)		

Covariates used in herptile models are described using the following abbreviations: “Ag”- Agriculture; “Wtr”- Water; “Wtl”- Wetland; “Grs”- Grassland; “Wod”- woodland; “1K”- 1,000m spatial scale; “500”- 500-m spatial scale; “200”- 200-m spatial scale; “PLND”- percentage of the landscape; “PD”- patch density; “ED” edge density; “LPI”- largest patch index; “Cld”- cloud cover (%); “Temp”- temperature (°C); “Wind”- wind speed (KM/hr); a covariate label of 1 means that there were no covariates modeled on the parameter.

Amphibian occupancy ranged from 0.04 (95% CI = 0.03, 0.05) for tiger salamander to 0.90 (95% CI = 0.898, 0.904) for northern leopard frog (*Lithobates pipiens*) (Table 1). Colonization probabilities for amphibians ranged from γ = 0.44 (95% CI = 0.36, 0.52) for northern leopard frog (*Lithobates pipiens*) to γ = 0.02 (95% CI = 0.019, 0.025) for tiger salamander (Table 1). Site occupancy probabilities for two of four frog species were most influenced by a negative effect of agriculture at the 1000-m scale (Table 2). Conversely, grasslands were the best predictor of site colonization for two frog species, although the effect was negative for southern leopard frog (*Lithobates sphenocephalus*). Landscape metrics for water and wetlands was the best predictor of either site occupancy or colonization for gray treefrog complex, southern leopard frog, and tiger salamander. Gray treefrog complex was the only amphibian to be show an effect to a woodland metrics, with a positive effect of the percentage of woodlands at the 1000-m scale on site occupancy (Table 2).

Reptiles generally had lower occupancy probabilities than amphibians, ranging from 0.01 (95% CI = -0.01, 0.03) for prairie ringneck snake (*Diadophis punctatus arnyi*) to 0.58 (95% CI = 0.48, 0.68) for common snapping turtle (*Chelydra serpentina*). Snake occupancy probabilities were best explained by agriculture and water metrics at various spatial scales. Western fox snake (*Pantherophis ramspotti*) was the most likely to colonize new areas (γ = 0.82, 95% CI = 0.62, 1.08), which was best predicted by a positive effect of water LPI at the 1000-m scale (Table 2). Agriculture and water landscape features were commonly correlated with occupancy and colonization probabilities for several snake species. The lone skink species studied, the prairie skink (*Plestiodon septentrionalis*), was positively associated with grassland features at the 1000-m scale for both occupancy and colonization (Table 2).

**Fig 2:**
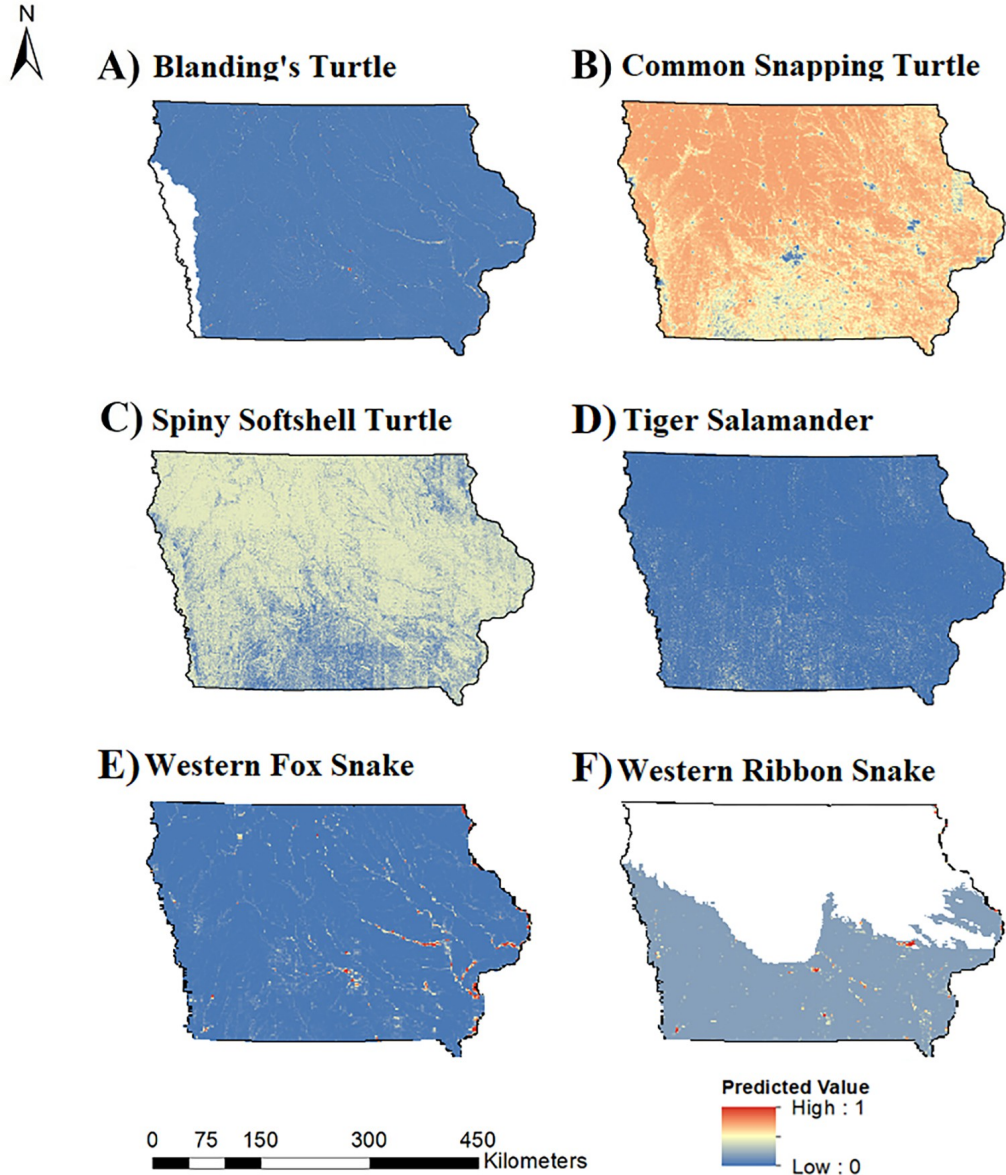
Predicted probability of occupancy for six herptile species of greatest conservation need (SGCN) in Iowa, 2006–2014. Maps display the predicted probability of occupancy in Iowa for A) Blanding’s turtle (*Emydoidea blandingii*), B) common snapping turtle (*Chelydra serpentina*), C) spiny softshell turtle (*Apalone spinifera*), D) tiger salamander (*Ambystoma tigrinum*), E) western fox snake (*Pantherophis ramspotti*), and F) western ribbon snake (*Thamnophis proximus*). Warm colors represent a high occupancy probability and cold colors represent low occupancy probability. For Blanding’s turtle (A) and western ribbon snake (F), predictions were restricted to the species’ ranges in Iowa. Basemap data provided by [66].

**Fig 3:**
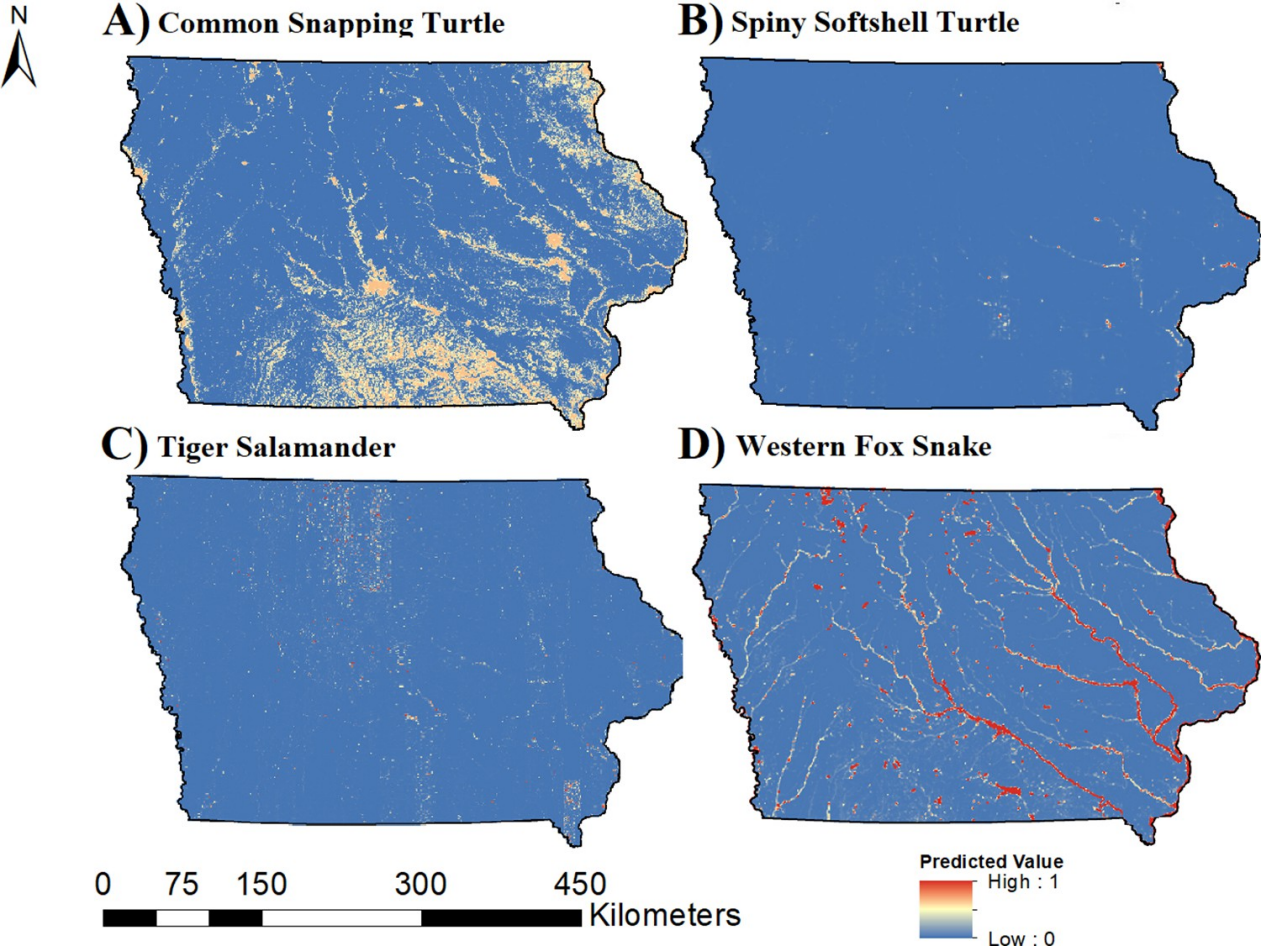
Predicted probability of colonization for four herptiles of greatest conservation concern in Iowa, 2006–2014. Maps display the predicted probability of colonization in Iowa for A) common snapping turtle (*Chelydra serpentina*), B) spiny softshell turtle (*Apalone spinifera*), C) tiger salamander (*Ambystoma tigrinum*), and western fox snake (*Pantherophis ramspotti*). Warm colors represent a high colonization probability and cold colors represent low colonization probability. Basemap data provided by [66].

**Fig 4:**
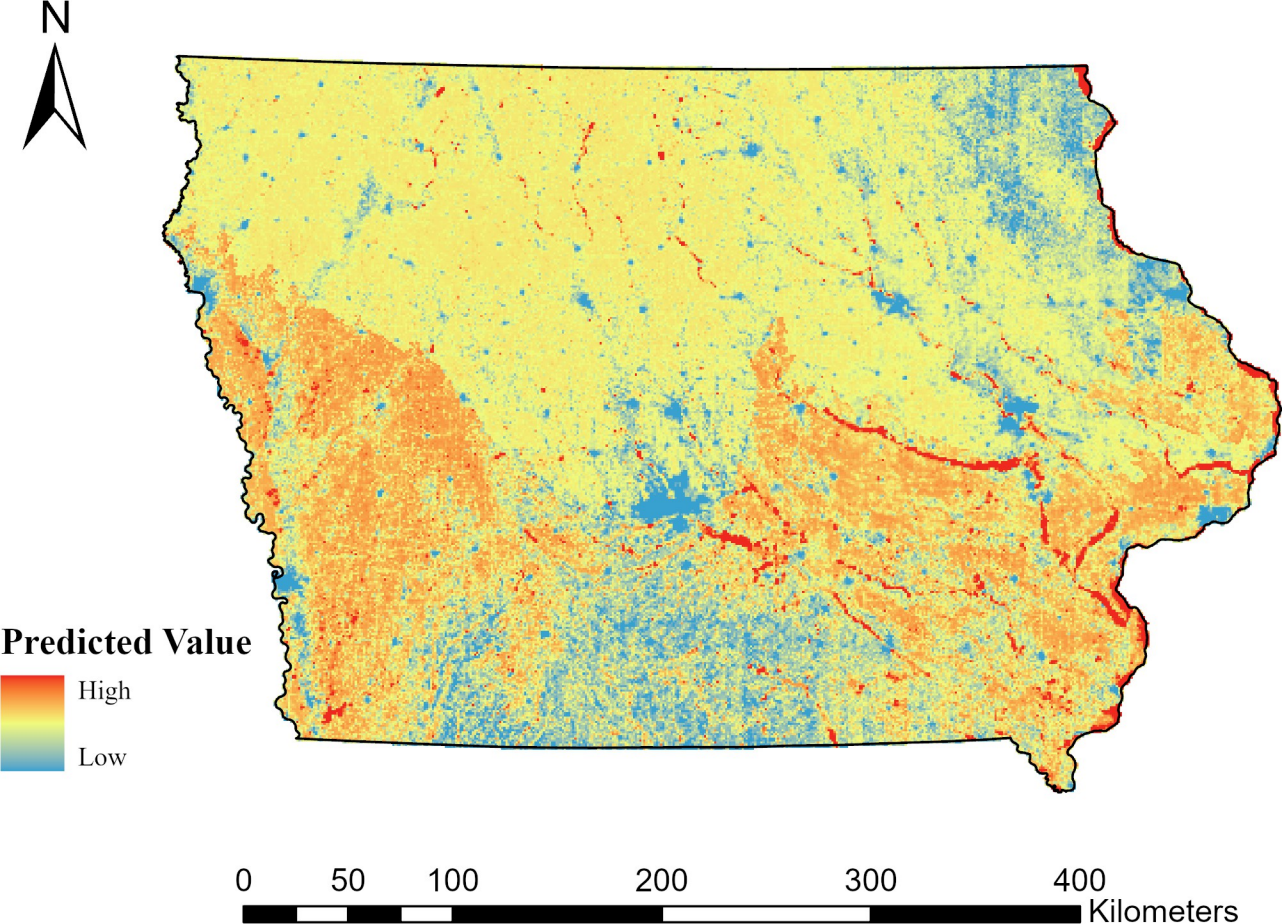
Cumulative predictive occupancy probability for six herptiles of greatest conservation need in Iowa, 2006–2014. The map displays the additive predicted occupancy probabilities of Blanding’s turtle (*Emydoidea blandingii*), common snapping turtle (*Chelydra serpentina*), spiny softshell turtle (*Apalone spinifera*), tiger salamander (*Ambystoma tigrinum*), western fox snake (*Pantherophis ramspotti*), and western ribbon snake (*Thamnophis proximus*) in Iowa. Warm colors represent high occupancy probabilities for multiple species and cold colors represent low occupancy probabilities. Basemap data provided by [66].

We considered predictive models for occupancy and colonization to be useful (AUC > 0.70) for six out of 15 species (40%), including all three turtle species, tiger salamander, western fox snake, and western ribbon snake (*Thamnophis proximus*). Predictive maps of occupancy (Fig 2) and colonization (Fig 3) probabilities for those species are provided. A cumulative map of herptile occupancy (Fig 4) based on the six aforementioned species suggests that hotspots are located in eastern and western Iowa, primarily along riparian areas.

## Discussion

Occupancy models across broad geographic areas are rare for herpetofauna, despite the importance of occupancy information to conservation and habitat management efforts [67]. By incorporating these model outputs into predictive maps across large spatial extents, conservation efforts and monitoring can be prioritized in areas with the highest probability of species occurrence. Spatially predictive models that account for detection probabilities decrease the likelihood of underestimating species’ distributions because occupancy probabilities are adjusted by survey-level detection covariates [1]. To our knowledge, our study is the first to provide such predictions of occupancy across a statewide landscape for many of these species. Below, we summarize our key findings by taxa and suggest how these predictions can be used to focus conservation efforts for herpetofauna.

### Amphibians

Wetland density has been found to increase amphibian occupancy due to species’ relatively small core ranges around a wetland base [68–70]. A high density of wetlands can increase variability of wetland characteristics, such as hydroperiods and emergent vegetation that have been found to affect occupancy of some amphibian species [71–74]. In our study, this was true for the southern leopard frog, where occupancy probability was best explained by water patch density at the 1000-m scale. We also found that tiger salamander colonization probability increased with wetland patch density. In Illinois, wetland connectivity and proximity has been shown to be an important metric to tiger salamander occupancy and colonization [75, 76] and patch density can be a useful indicator of habitat connectivity in a metapopulation [77]. Our predictions on tiger salamander colonization probability were low across Iowa, but we found high probabilities within specific regions of the state, such as the Prairie Pothole Region (PPR) in north central Iowa. However, due to agriculture practices and changing weather patterns, the wetter regions of the state, such as the PPR, are under threat [78, 79]. Increasing wetland variability has been shown to alter metapopulation dynamics of amphibians that rely on wetlands to breed, where spatiotemporal variation in wetland hydrology can alter structural and functional connectivity of wetland patches [79]. To combat these stressors on amphibians and support occupancy and colonization, increasing wetland density and wetland size could provide the variation in wetland characteristics to support occupancy and colonization of new areas [80]. Since the 1990s, funds from the Wetland Reserve Program (WRP) has increased the area of wetlands in Iowa by approximately 5,000 ha, which had been converted into agricultural development [16]. These restored wetlands have been shown to have occupancy probabilities ranging from 81–100% for frogs and toads, where wetland colonization was faster when restored wetland were close to source wetlands [81].

Although wetlands are often considered the most important habitat factor for the occupancy of amphibian species, upland habitat can be just as critical [82–84]. Agricultural development may limit the movement between populations, thereby negatively affecting occupancy of northern leopard frog and Blanchard’s cricket frog. For example, Swanson et al. [24] found fewer telemetry locations of northern leopard frog in agricultural patches compared to adjacent grasslands and wetlands. However, the influence of agricultural practices and types of agriculture on occupancy and habitat use may be conditional on agricultural intensity [85, 86]. Lower intensity practices, such as cattle grazing or cover crops, have shown to improve occupancy as compared to high-intensity row cropping practices [85, 86]. While some species, such as the American toad, can use row crops in the late growing season [87], we found that the top model for four amphibian species included a negative effect of agriculture. Consequently, broad-scale conversion of native uplands into agriculture has likely limited the dispersal ability of several amphibian species of greatest conservation need statewide. Our findings suggest that conserving remaining grasslands and wetlands or restoring such areas can support the colonization of species like the Blanchard’s cricket frog. Additionally, grasslands can provide cover that can aid in reducing desiccation and predation, thereby allowing amphibians to disperse more easily [88].

### Reptiles

Similar to amphibians, agriculture was the most important landscape feature affecting the occupancy of reptiles. This relationship was negative for four of these species, whereas Blanding’s turtle exhibited a positive relationship with agriculture. Many turtles, including Blanding’s, nest during May and June [58] when corn and soybean fields often have little to no vegetative growth. Turtles are often attracted to cultivated land for nesting because they seek out open-canopied habitats that allow subterranean nests adequate warmth to facilitate embryonic development [89–91]. However, nesting in agriculture fields can be an ecological trap because corn and soybeans often grow to heights that shade out nests, making the areas too cool for adequate development [91, 92]. Additionally, given the relatively long lifespan of Blanding’s turtles [58], occupancy in agricultural areas may result from remnant populations in new development. Whether Blanding’s turtle occupancy is actually higher in large patches of agriculture warrants further study. As for the four species of reptiles that exhibited a negative association with agriculture, this likely stems from a lack of diversity of microhabitats, flora, and fauna, and consequently, food resources in agricultural landscapes [93–95]. Additionally, agricultural chemicals can harm developing young [92] and kill invertebrates that lizards and juvenile snakes feed upon, crops can block visual cues that reptiles need to orient [95], and machinery likely kills reptiles during tilling, planting, chemical application, and harvesting [96]. Two species, northern watersnake and common snapping turtle, showed negative effects of agriculture on colonization probabilities. Although common snapping turtle are considered relatively tolerant of agriculture due to their common nesting forays into agriculture fields [92], our results suggest such presence is probably not indicative of preference.

Woodlands had a negative effect on common snapping turtle occupancy and were included in a negative interaction with wetlands in the top model for northern redbelly snake colonization. Like Blanding’s turtle, common snapping turtles construct subterranean nests to oviposit eggs, and the temperature of the nest influences both somatic and sexual development [91, 97]. A lack of open-canopied habitats that are preferred for nesting could contribute to our finding that forested areas negatively affect their occupancies. However, for some woodland specialists, such as northern redbelly snake, woodland edges along rivers and streams can act as travel corridors in Iowa’s agricultural matrix. Woodlands in Iowa have decreased since the mid 1800’s at the state scale [98]; however, forest cover has increased in Iowa’s urban areas [99]. We found occupancy probabilities of common snapping turtle decreased in urban areas. This is most apparent in the spatial predictions of common snapping turtle occupancy probability, where areas of lowest probabilities are in Des Moines and Cedar Rapids regions. However, the predicted colonization probabilities in urban areas were comparatively high for common snapping turtles, likely due to limited agricultural development in these areas. This contradiction illustrates the difficulty in interpreting the effect of urbanization on species, given that urbanization often correlates with other landcover types.

Water bodies and wetlands had species-specific effects on occupancy or colonization for seven species of reptiles coinciding with their unique ecologies. For example, western fox snake, which is commonly found near wetland edges [58], had a higher occupancy probability in areas with higher percentages of water; whereas plains garter snake, an upland specialist, had decreased occupancy probabilities in areas with high water LPI. Colonization probabilities were negatively affected by water and wetlands for two reptile species. Such negative relationships on colonization may represent tradeoff with preferred habitat. For example, wetland patch density decreased colonization of plains garter snake and water area decreased colonization of prairie ringneck snake. Plains garter snakes prefer upland habitat and prairie ringneck snakes prefer rocky terrestrial areas [58], which are both precluded by wetlands and water. In contrast, positive landscape associations likely represent greater connectivity with preferred habitat, such as spiny softshell colonization increasing with water patch density and western fox snake colonization increasing with water LPI.

Threats to wetlands in the PPR and native grasslands in the midwestern United States pose increased risk to the viability of species that rely on those habitats [99–102]. Changing weather patterns are predicted to reduce wetland productivity throughout the PPR [103], which will likely decrease habitat for wetland-dependent species. This is particularly true for wetlands in the northern and western portion of the PPR, which are at greater risk of drying and instability due to drought [103]. The eastern border of the PPR, including Iowa’s Des Moines Lobe ecoregion, may be a potential climatic refuge for wetlands [103] and wetlands edges. However, this refuge potential may be limited due to the widespread conversion of wetlands and grasslands to agriculture in Iowa [103]. Since the mid-1800s, almost 75% of Iowa’s grasslands have been converted into agriculture statewide and the majority of wetlands in the Des Moines lobe ecoregion have been drained [98], thereby restricting large patches of wetlands and grasslands. Grasslands in Iowa face an additional threat of woody encroachment [104], which poses a strong threat to the current abundances of reptiles but may increase some amphibian abundances [102]. For example, in northwest Indiana, a gradient of open grasslands to forest corresponded to a decrease in reptile abundances but increases in amphibian abundances [102]. Consequently, weather and broad-scale conversion of wetlands and grasslands likely pose the greatest risks to habitat for many SGCN herptiles, potentially resulting in community composition shifts. Future habitat management and protection efforts in Iowa should prioritize areas with the highest probability of species’ occupancy and colonization probabilities to increase the efficiency of efforts. Our analyses of cumulative occupancy probabilities indicate that these regions primarily include large water bodies, such as the Iowa River, Mississippi River, and Lake Red Rock, and their adjacent uplands.

## Conclusion

Spatial predictions of occupancy and colonization probabilities help land managers target potential areas for surveys, habitat management, and restoration efforts [105]. One obvious benefit to occupancy predictions is the ability to identify locations where SGCN may be present but have not previously been documented. Survey effort and resources can be targeted in these locations, which can inform and update species’ ranges and habitat management plans. Predictions for species’ colonization probabilities provide land managers with information on landscape features that attract individuals but those predictions may be missing additional attributes to maintain a population. Consequently, habitat restoration efforts can be focused on shifting characteristics to increase occupancy probabilities and connectivity. Spatial estimates of colonization probabilities can also provide insight into metapopulation dynamics [106, 107]. Metapopulation theory predicts that larger, more connected patches have the greatest potential for colonization [108–110]. Our models support this for Blanchard’s cricket frog, spiny softshell turtle, and tiger salamander; colonization probabilities of these species were best predicted by a positive effect of patch density.

We sought to estimate the effect of agricultural development on herptile metapopulation dynamics and to provide species-specific predictions of occupancy and colonization for land managers in agriculturally dominated landscape. Perhaps the most common finding among all species was a negative effect of agriculture on either occupancy or colonization probabilities, where seven out of 15 species (47%) included a negative effect of agriculture in the top model. Although agricultural development in Iowa is unlikely to significantly decrease due to socio-economic reasons, habitat conservation efforts, such as the WRP, Conservation Reserve Program (CRP), and prairie strips, may provide habitat refugia or travel corridors necessary to maintain populations. The importance of WRP and CRP to herptiles in agricultural landscapes have been documented [111–113]. Similarly, the implementation of prairie strips in Iowa have been shown to increase diversity of invertebrates and birds [114], but the effects on herpetofauna have not been studied. As agricultural development continues to expand in Iowa and the midwestern United States, the implementation of such conservation efforts in conjunction with agricultural lands will likely become increasingly important to sustaining herptile communities.

## Supporting information

S1 Table Public properties sampled for herptiles. Properties with associated habitat types, ownership, county, and year in which they were surveyed for herptiles.
(XLSX)

S2 Table Range of values for landscape-level habitat variables. (XLSX)

S3 Table Correlation matrix of landscape-level habitat variables. Dark blue colors show strong negative correlation and dark green colors show strong positive correlation. Variables with a Pearson correlation coefficient > 0.7 were not included within the same model.
(XLSX)

S4 Table Justification for inclusion of covariate interactions to predict occupancy (Ψ) for gray treefrog complex (*Hyla* spp.) and northern redbelly snake (*Storeria occipitomaculata*). For each species, interactions were included at every scale and with every combination of covariates fitted to colonization probability (γ) and detection probability (p).
(DOCX)

## References

[1] MacKenzie DI, Nichols JD, Lachman GB, Droege S, Royle JA, Langtimm CA. Estimating site occupancy rates when detection probabilities are less than one. Ecology. 2002;83:2248–2255.

[2] Broton L, Thuiller W, Araújo MB, Hirzel AH. Presence-absence versus presence-only modelling methods for predicting bird habitat suitability. Ecography. 2004;27:437–448.

[3] Schoch GC, Dethier MD. Scaling up: the statistical linkage between organismal abundance and geomorphology on rocky intertidal shorelines. J Exp Mar Biol Ecol. 1996;201:37–72.

[4] Angermeier PL, Winston MR. Characterizing fish community diversity across Virginia landscapes: prerequisite for conservation. Eco App. 1999;9:335–349.

[5] Whittaker RJ, Araújo MB, Jepson P, Ladle RJ, Watson JEM, Willis KJ. Conservation biogeography: assessment and prospect. Diversity Distrib. 2005;11:3–23.

[6] Baldwin RF, Colhoun AJK, deMaynadier PG. Conservation planning for amphibian species with complex habitat requirements: a case study using movements and habitat selection of the wood frog *Rana sylvatica*. J Herpetol. 2006;40:442–453.

[7] Gilioli KG, Kéry M, Guimarães. Unraveling fine-scale habitat use for secretive species: when and where toads are found when not breeding. PLoS ONE. 2018;13:e0205304 doi: 10.1371/journal.pone.0205304 30296275 PMC6175507

[8] Gibbons JW, Scott DE, Ryan TJ, Buhlmann KA, Tuberville TD, Metts BS, et al. The global decline of reptiles, déjà vu amphibians. BioScience. 2000;50:653–666.

[9] Watling JI, Nowakowski AJ, Donnelly MA, Orrock JL. Meta-analysis reveals the importance of matrix composition for animals in fragmented habitat. Glob Ecol Biogeogr. 2011;20:209–217.

[10] Baldwin RF, Calhoun AJK, deMaynadier PG. The significance of hydroperiod and stand maturity for pool-breeding amphibians in forested landscapes. Can J Zool. 2006;84:1604–1615.

[11] Blevins E, With KA. Landscape context matters: local habitat and landscape effects on the abundance and patch occupancy of collared lizards in managed grasslands. Lands Ecol. 2007;26:837–850.

[12] Laurance WF, Useche DC, Rendeiro J, Kalka M, Bradshaw CJA, Sloan SP, et al. Averting biodiversity collapse in tropical forest protected areas. Nature. 2012;489:290–294. doi: 10.1038/nature11318 22832582

[13] de Oliveira-Junior ND, Heringer G, Bueno ML, Pontara V, Meira-Neto JAA. Prioritizing landscape connectivity of a tropical forest biodiversity hotspot in global change scenario. For Ecol Manage. 2020;472:118247.

[14] Matos FAR, Magnago LFS, Miranda CAC, de Menezes LFT, Gastauer M, Safar NVH. Secondary forest fragments offer important carbon and biodiversity cobenefits. Glob Chang Biol. 2020;26:509–522. doi: 10.1111/gcb.14824 31486174

[15] Bishop RA. Iowa’s wetlands. Proc Iowa Acad Sci. 1981;88:11–16.

[16] Iowa Department of Natural Resources. Iowa Wildlife Action Plan: securing a future for fish and wildlife–a conservation legacy for Iowans. Iowa Department of Natural Resources, Des Moines, USA; 2015

[17] Bultena GL, Duffy MD, Jungst SE, Kanwar RS, Menzel BW, Misra MK, et al. Effects of agricultural development on biodiversity: a lesson from Iowa. Economics Technical Reports and White Papers. 1996;41.

[18] Vié JC, Hilton-Taylor C, Stuart SN. Wildlife in a changing world–an analysis of the 2008 IUCN Red List of threatened species. IUCN, Switzerland; 2009.

[19] Christoffel RA, Lepczyk CA. Representation of herpetofauna in wildlife research journals. J Wildl Manage. 2012;76:661–669.

[20] Titley MA, Snaddon JL, Turner EC. Scientific research on animal biodiversity is systematically biased towards vertebrates and temperate regions. PLoS ONE. 2017;12: e0189577. doi: 10.1371/journal.pone.0189577 29240835 PMC5730207

[21] MacNally R, Brown GW. Reptiles and habitat fragmentation in the box-ironbark forests of central Victoria, Australia: predictions, compositional change and faunal nestedness. Oecologia. 2001;128:116–125. doi: 10.1007/s004420100632 28547081

[22] Driscoll DA. Extinction and outbreaks accompany fragmentation of a reptile community. Eco App. 2004;14:220–240.

[23] Lesbarrères D, Ashpole SL, Bishop CA, Blouin-Demers G, Brooks RJ, Echaubard P, et al. Conservation of herpetofauna in northern landscapes: threats and challenges from a Canadian perspective. Biol Conserv. 2014;48–55.

[24] Swanson JE, Muths E, Pierce CL, Dinsmore SJ, Vendever MW, Hladik ML, et al. Exploring the amphibian exposome in an agricultural landscape using telemetry and passive sampling. Sci Rep. 2018;8:10045. doi: 10.1038/s41598-018-28132-3 29968741 PMC6030078

[25] Swanson JE, Pierce CL, Dinsmore SJ, Smalling KL, Vandever MW, Stewart TW, et al. Factors influencing anuran wetland occupancy in an agricultural landscape. Herpetologica. 2019;75:47–56.

[26] Thompson CM, Sweeney MR, Popescu VD. Carryover effects of pesticide exposure and pond drying on performance, behavior, and sex ratios in a pool breeding amphibian. J Zool. 2022;317:229–240.

[27] Relyea RA. Predator cues and pesticides: a double dose of danger for amphibians. Eco App. 2003;13:1515–1521.

[28] Hanlon SM, Relyea R. Sublethal effects of pesticides on predator-prey interactions in amphibians. Ichthyol Herpetol. 2013;2013:691–698.

[29] Cushman SA. Effects of habitat loss and fragmentation on amphibians: a review and prospectus. Biol Conserv. 2006;128:231–240.

[30] Moore JA, Gillingham JC. Spatial ecology and multi-scale habitat selection by a threatened rattlesnake: The Eastern Massasauga (Sistrurus catenatus catenatus). Copeia 2006;2006:742–751.

[31] Hoss SK, Guyer C, Smith LL, Schuett GW. Multiscale influences of landscape composition and configuration on the spatial ecology of Eastern Diamond-backed Rattlesnakes (Crotalus adamanteus). J Herpetol. 2010;44:110–123.

[32] Baxley D, Lipps GJ, Qualls CP. Multiscale habitat selection by Black Pine Snakes (Pituophis melanoleucus lodingi) in southern Mississippi. Herpetologica. 2011;67:154–166.

[33] Dodd CK, Cade BS. Movement patterns and the conservation of amphibians breeding in small, temporary wetlands. Conserv Biol. 1998;12:331–339.

[34] Harvey DS, Weatherhead PJ. A test of the hierarchical model of habitat selection using Eastern Massasauga Rattlesnakes (Sistrurus c. catenatus). Biol Conserv. 2006;130:206–216.

[35] Steen DA, Linehan JM, Smith LL. Multiscale habitat selection and refuge use of Common Kingsnakes, *Lampropeltis getula*, in southwest Georgia. Copeia 2010;2010:227–231.

[36] Steen DA, Godwin JC, McClure CJW, Barbour M. Informing management of endemic habitat specialists: Multiscale habitat selection by the Red Hills Salamander. J Wildl Manag 2014;78:463–470.

[37] Marchand MN, Litvaitis JA. Effects of habitat features and landscape composition on the population structure of a common aquatic turtle in a region undergoing rapid development. Conserv Biol. 2004;18:758–797.

[38] Mazerolle MJ, Desrochers A, Rochefort L. Landscape characteristics influence pond occupancy by frogs after accounting for detectability. Eco App. 2005;15:824–834.

[39] Weir LA, Royle JA, Nanjappa P, Jung RE. Modeling anuran detection and site occupancy on North American Amphibian Monitoring Program (NAAMP) routes in Maryland. J Herpetol. 2005;39:627–639.

[40] Steen DA, McClure CJW, Brock JC, Rudolph DC, Pierce JB, Lee JR, et al. Landscape-level influences of terrestrial snake occupancy within the southeastern United States. Eco App. 2012;22:1084–1097. doi: 10.1890/11-1777.1 22827120

[41] Scherer RD, Muths E, Noon BR. The importance of local and landscape-scale processes to the occupancy of wetlands by pond-breeding amphibians. Popul Ecol. 2012;54:487–498.

[42] Larson DM. Grassland fire and cattle grazing regulate reptile and amphibian assembly among patches. Enviro Manag. 2014;54:1434–1444. doi: 10.1007/s00267-014-0355-2 25156864

[43] Paterson JE, Pulfer T, Horrigan E, Sukumar S, Vezina BI, Zimmerling R, et al. Individual and synergistic effects of habitat loss and roads on reptile occupancy. Glo Eco Con. 2021;31:e01865.

[44] Michael DR, Ikin K, Crane M, Okada S, Lindenmayer DB. Scale-dependent occupancy patterns in reptiles across topographically different landscapes. Ecography. 2017;40:415–424.

[45] Prieto-Ramirez AM, Rӧhler L, Cord AF, Pe’er G, Rӧdder D, Henle K. Differential effects of habitat loss on occupancy patterns of the eastern green lizard Lacerta viridis at the core and periphery of its distribution range. PLoS ONE. 2020;15: e0229600. doi: 10.1371/journal.pone.0229600 32134932 PMC7058328

[46] Royal EJ, Kross CS, Wilson JD. Legacy land use predicts occupancy patterns of prairie-associated herpetofauna in Western Arkansas. Landsc Ecol. 2023;38:423–438. 10.1007/s10980-022-01564-z.

[47] MacKenzie DI, Nichols JD, Hines JE, Knutson MG, Franklin AB. Estimating site occupancy, colonization, and local extinction when a species is detected imperfectly. Ecology. 2003;84:2200–2207.

[48] Chandler RB, Muths E, Sigafus BH, Schwalbe CR, Jarchow CJ, Hossack BR. Spatial occupancy models for predicting metapopulation dynamics and viability following reintroduction. J Appl Ecol. 2015;52:1325–1333.

[49] Prior J. Landforms of Iowa [Internet]. Iowa City, IA: University of Iowa Press; 1991. Available: http://sustainableag.unl.edu/pdf/landformsofiowacari.pdf.

[50] Iowa Department of Natural Resources. Iowa Wildlife Action Plan: securing a future for fish and wildlife–a conservation legacy for Iowans. Iowa Department of Natural Resources, Des Moines, USA; 2006.

[51] Kinkead KE. Iowa Multiple Species Inventory and Monitoring Program Technical Manual. Des Moines, IA: Iowa Department of Natural Resources. 2016. Available from: https://www.iowadnr.gov/Conservation/Iowas-Wildlife/Wildlife-Diversity-Program/Diversity-Projects.

[52] Environmental Services Research Institute (ESRI). ArcGIS ver. 10.1. 2012 [Accessed 11 Mar 2013]. Available from: http://www.esri.com.

[53] Grant WG, Anton DT, Lovich JE, Mills AE, Philip PM, Gibbons JW. The use of coverboards in estimating patterns of reptile and amphibian biodiversity, in: McCullough D.R., and Barrett R.H. (Eds.), Wildlife 2001: Populations. Elsevier Science Publishers Ltd., London, pp. 379–403; 2002.

[54] Bennett RM, Ross RM, Lellis WA, Redell LA. Terrestrial salamander preference for artificial cover objects made from four species of wood. J Penn Acad Sci. 2003;76:77–79.

[55] Halliday WD, Blouin-Demers G. Efficacy of coverboards for sampling small northern snakes. Herpetol Notes. 2015;8:309–314.

[56] Lemm JM, Tobler MW. Factors affecting the presence and abundance of amphibians, reptiles, and small mammals under artificial cover in southern California. Herpetologica. 2021;77:307–319.

[57] LeClere JB. A field guide to the amphibians and reptiles of Iowa. Ecouniverse, Rodeo, New Mexico; 2014.

[58] Beyer HL. Geospatial Modelling Environment ver. 0.7.2.1. http://www.spatialecology.com/gme. 2012; Accessed 13 March 2013.

[59] McGarigal K, Cushman SA, Neel MC. Ene E FRAGSTATS: Spatial Pattern Analysis Program for Categorical Maps. Computer software program produced by authors, University of Massachusetts, Amherst. 2002 [Accessed 13 March 2013]. Available from: http://www.umass.edu/landeco/research/fragstats/fragstats.htm.

[60] White GC, Burnham KP. Program MARK: Survival estimation from populations of marked animals. Bird Study 1999;46:120–139.

[61] Olson GS, Anthony RG, Forsman ED, Ackers SH, Loschl PJ, Reid JA, et al. Modeling of site occupancy dynamics for Northern Spotted Owls, with an emphasis on the effects of Barred Owls. J Wildl Manag. 2005;69:918–932.

[62] Burnham KP, Anderson DR. Model Selection and Multimodal Inference: A Practical Information-theoretic Approach. Springer-Verlag, New York; 2002.

[63] Richards SA, Whittingham MJ, Stephens PA. Model selection and model averaging in behavioural ecology: the utility of the IT-AIC framework. Behav Ecol Sociobiol. 2011;65:77–89.

[64] National Park Service. NPS AlaskaPak ver. 3.0. 2010 [Accessed 13 March 2013]. Available from: http://science.nature.nps.gov/im/gis/alaskapak.cfm.

[65] Swets KA. Measuring the accuracy of diagnostic systems. Science. 1988;240:1285–1293. doi: 10.1126/science.3287615 3287615

[66] Iowa DNR NRGIS. 2024. Database: Iowa Geospatial Data Clearinghouse. Available from: https://geodata.iowa.gov/.

[67] Evans AM, Marshal JP, Alexander GJ. Forest patch characteristics affect reptile occurrence in north-western Madagascar. Austral Ecol. 2021;46:424–436.

[68] Gibbs JP. Wetland loss and biodiversity conservation. Conserv Biol. 2000;14:314–317. 10.1046/j.1523-1739.2000.98608.x.

[69] Semlitsch RD, Bodie JR. Biological criteria for buffer zones around wetlands and riparian habitats for amphibians and reptiles. Conserv Biol. 2003;17:1219–1228. 10.1046/j.1523-1739.2003.02177.x.

[70] Rittenhouse TAG, Semlitsch RD. Distribution of amphibians in terrestrial habitat surrounding wetlands. Wetlands. 2007;27:153–161.

[71] Reeves RA, Pierce CL, Smalling KL, Klaver RW, Vandever MW, Battaglin WA, et al. Restored Agricultural Wetlands in central Iowa: Habitat Quality and Amphibian Response. Wetlands. 2016;36:101–110. 10.1007/s13157-015-0720-9.

[72] Hamer AJ, Schmera D, Mahony MJ. Multi‐species occupancy modeling provides novel insights into amphibian. Ecol Appl. 2021;31:e2293. 33432692 10.1002/eap.2293

[73] Buckardt EM. Amphibian occupancy and diversity on a post-mined landscape MS Thesis. Pittsburg State University. 2022. Available from: https://digitalcommons.pittstate.edu/cgi/viewcontent.cgi?article=1517&context=etd.

[74] Buckardt EM, Rega-Brodsky CC, George AD. Post-mined wetlands provide breeding habitat for amphibians. Wetlands. 2023;43:75. 10.1007/s13157-023-01720-4.

[75] Cosentino BJ, Schooley RL, Phillips CA. Spatial connectivity moderates the effect of predatory fish on salamander metapopulation dynamics. Ecosphere. 2011;2:1–14.

[76] Vanek JP, King RB, Glowacki GA. Landscape and management factors influence the occupancy dynamics of sympatric salamanders in an urban preserve system. Glob Ecol Conserv. 2019;20:e00742.

[77] Frey SJK, Strong AM, McFarland KP. The relative contribution of local habitat and landscape context to metapopulation processes: a dynamic occupancy modeling approach. Ecography. 2012;35:581–589.

[78] Rashford BS, Adams RM, Wu J, Voldseth RA, Guntenspergen GR, Werner B, et al. Impacts of climate change on land-use and wetland productivity in the Prairie Pothole Region of North America. Reg Environ Change. 2016;16:515–526.

[79] Johnson WC, Werner B, Guntenspergen GR, Voldseth RA, Millett B, Naugle DE, et al. Prairie wetland complexes as landscape functional units in a changing climate. BioScience. 2010;60:128–140.

[80] Bertassello LE, Jawitz JW, Bertuzzo E, Botter G, Rinaldo A, Aubeneau AF, et al. Persistence of amphibian metapopulation occupancy in dynamic wetlandscapes. Landsc Ecol. 2022;37:695–711. 10.1007/s10980-022-01400-4.

[81] Bartelt PE, Klave RW. Response of Anurans to Wetland Restoration on a Midwestern Agricultural Landscape. J Herpet. 2017;51:504–514.

[82] Scherer RD, Muths E, Noon BR. The importance of local and landscape-scale processes to the occupancy of wetlands by pond-breeding amphibians. Popul Ecol. 2012;54:487–498. 10.1007/s10144-012-0324-7.

[83] Sawatzky ME, Martin AE, Fahrig L. Landscape context is more important than wetland buffers for farmland amphibians. Agric Ecosyst Environ. 2019;269:97–106. 10.1016/j.agee.2018.09.021.

[84] Howell HJ, Mothes CC, Clements SL, Catania SV, Rothermel BB, Searcy CA. Amphibian responses to livestock use of wetlands: New empirical data and a global review. Ecol Appl 2019;29:e01976. doi: 10.1002/eap.1976 31323162

[85] Hromada SJ, Iacchetta MG, Beas BJ, Flaherty J, Fulbright MC, Wild KH, et al. Low-intensity agriculture shapes amphibian and reptile communities: insights from a 10-year monitoring study. Herpetologica. 2021;77:294–306.

[86] Koumaris A, Fahrig L. Different anuran species show different relationships to agricultural intensity. Wetlands. 2016;36:731–744. 10.1007/s13157-016-0781-4.

[87] Collins SJ, Fahrig L. Responses of anurans to composition and configuration of agricultural landscapes. Agric Ecosyst Environ. 2017;239:399–409. 10.1016/j.agee.2016.12.038.

[88] Bartelt PE, Klaver RW. Response of anurans to wetland restoration on a midwestern agricultural landscape. J Herpet. 2017;51:504–514. 10.1670/16-113.

[89] Balas CJ, Euliss NH, Mushet DM. Influence of conservation programs on amphibians using seasonal wetlands in the prairie pothole region. Wetlands, 2012;32:333–345. 10.1007/s13157-012-0269-9.

[90] Castellano CM, Behler JL, Ultsch GF. Terrestrial movements of hatchling wood turtles (Glyptemys insculpta) in agricultural fields in New Jersey. Chelonian Conserv Biol. 2008;7:113–118.

[91] Refsnider JM, Janzen FJ. Putting eggs in one basket: ecological and evolutionary hypotheses for variation in oviposition-site choice. Annu Rev Ecol Evol Syst. 2010;41:39–57.

[92] Freedberg S, Lee C, Pappas M. Agricultural practices alter sex ratio in a reptile with environmental sex determination. Biol Conserv. 2011;144:1159–1166.

[93] Thompson M, Coe BH, Andrews RM, Cristol DA, Crossley DA II, Hopkins WA. Agricultural land use creates evolutionary traps for nesting turtles and is exacerbated by mercury pollution. J Exp Zool. 2017;329:230–243. 10.1002/jez.2198 29962084

[94] Liebman M, Helmers MJ, Schulte LA, Chase CA. Using biodiversity to link agricultural productivity with environmental quality: Results from three field experiments in Iowa. Renew Agric Food Syst. 2013;28:115–128.

[95] Pappas MJ, Congdon JD, Brecke BJ, Freedberg S. Orientation of freshwater hatchling Blanding’s (Emydoidea Blandingii) and snapping turtles (Chelydra serpentina) dispersing from experimental nests in agricultural fields. Herpetol Conserv Biol. 2013;8:385–399.

[96] Fischer B, Lamey A. Field deaths in plant agriculture. J Agric Environm Ethics. 2018;31:409–428.

[97] Krueger CJ, Janzen FJ. On the origin of patterns of temperature-dependent sex determination. Evolution. 2023;77:1091–1100. doi: 10.1093/evolut/qpad029 36801993

[98] Gallant AL, Sadinski Roth MF, Rewa CA Changes in historical Iowa land cover as context for assessing the environmental benefits of current and future conservation efforts on agricultural lands. J Soil Water Conserv; 2011;66:67–77.

[99] Bowman TA, Thompson JR, Tyndall JC, Anderson PF. Land cover analysis for urban foresters and municipal planners: examples from Iowa. J Forestry; 2011;110:23–33.

[100] Doherty KE, Ryba AJ, Stemler CL, Niemuth ND, Meeks WA. Conservation planning in an era of change: state of the U.S. prairie pothole region. Wildl Soc Bul. 2013;37: 546–563.

[101] Niemuth ND, Fleming KK, Reynolds RE. Waterfowl conservation in the us prairie pothole region: confronting the complexities of climate change. PLoS ONE. 2014;9:e100034. doi: 10.1371/journal.pone.0100034 24937641 PMC4061047

[102] Grundel R, Beamer DA, Glowacki GA, Frohnapple KJ, Pavlovic NB. Opposing responses to ecological gradients structure amphibian and reptile communities across a temperate grassland-savanna-forest landscape. Biodivers Conserv. 2015;24:1089–1108.

[103] Rashford BS, Adams RM, Wu J, Voldseth RA, Guntenspergen GR, Werner B, et al. Impacts of climate change on land-use and wetland productivity in the Prairie Pothole Region of North America. Reg Environ Change. 2016;16:515–526.

[104] Harr RN, Morton LW, Rusk SR, Engle DM, Miller JR, Debinski D. Landowner’s perceptions of risk in grassland management: woody plant encroachment and prescribed fire. Ecol Soc. 2014;19:41.

[105] Harms TM, Murphy KT, Lyu X, Patterson SS, Kinkead KE, Dinsmore SJ, et al. Using landscape habitat associations to prioritize areas of conservation action for terrestrial birds. PLoS ONE. 2017;12:e0173041. doi: 10.1371/journal.pone.0173041 28301877 PMC5354636

[106] Ozgul A, Armitage KB, Blumstein DT, VanVuren DH, Oli MK. Effects of patch quality and network structure on patch occupancy dynamics of a yellow-belled marmot metapopulation. J Ani Eco. 2006;75:191–202. 10.1111/j.1365-2656.2006.01038.x 16903056

[107] Alverson KM, Dinsmore SJ. Factors affecting burrowing owl occupancy of prairie dog colonies. PLoS ONE. 2014;116:242–250.

[108] Hanski I. A practical model of metapopulaion dynamics. J Anim Ecol. 1994;63:151–162.

[109] Thomas CD. Extinction, colonization, and metapopulations: environmental tracking by rare species. Conserv Biol. 1994;8:373–378.

[110] Hanski I. Metapopulation dynamics. Nature. 1998;396:41–49.

[111] Behrman KD, Juenger TE, Kiniry JR, Keitt TH. Spatial land use trade-offs for maintenance of biodiversity, biofuel, and agriculture. Landsc Ecol. 2015;30:1987–1999.

[112] Waddle JH, Glorioso BM, Faulkner SP. A quantitative assessment of the conservation benefits of the wetlands reserve program to amphibians. Restor Ecol. 2012;21:200–206.

[113] Walls SC, Waddle JH, Faulkner SP. Wetland reserve program enhances site occupancy and species richness in assemblages of Anuran amphibians in the Mississippi Alluvial Valley, USA. Wetlands. 2013;34:197–207.

[114] Schulte LA, Niemi J, Helmers MJ, et al. Prairie strips improve biodiversity and the delivery of multiple ecosystem services from corn-soybean croplands. PNAS. 2017;114:11247–11252. doi: 10.1073/pnas.1620229114 28973922 PMC5651729

