# Supplementary material for: Broad-scale predictions of herpetofauna occupancy and colonization in an agriculturally dominated landscape
Source: PLoS One. 2024 Oct 30;19(10):e0306655. doi: 10.1371/journal.pone.0306655 (PMC11524447; doi:10.1371/journal.pone.0306655)
Supplement: S4 Table — For each species, interactions were included at every scale and with every combination of covariates fitted to colonization probability (γ) and detection probability (p). (DOCX) [file pone.0306655.s004.docx]

**S4. Table. Justification for inclusion of covariate interactions to predict occupancy (Ψ) for gray treefrog complex (*Hyla spp.*) and northern redbelly snake (*Storeria occipitomaculata*).** For each species, interactions were included at every scale and with every combination of covariates fitted to colonization probability (γ) and detection probability (p).

| Species | Model Interaction | Justification |
| --- | --- | --- |
| Hyla spp | Ψ (~WodPLND * WtrPLND) γ (~1) p(~1) | "They prefer moderate to heavily wooded habitats…Large populations are often centered in river valleys" LeClere 2014 p. 83 (Referencing Eastern Graytree Frog) |
|  | Ψ (~WodED*GrsED) γ (~1) p(~1) | "They inhabit the environs of…open grasslands, oak savannas, and forst edges" LeClere 2014, p. 80 (Referencing Cope's Graytree Frog) |
|  | Ψ (~GrsPLND*WodPLND) γ (~1) p(~1) | Described as "a prairie or oak savanna species" Oldfield et al. 1994 |
|  | Ψ (~GrsPLND * WtrPLND) γ (~1) p(~1) | "They inhabit wetlands in prairies…"LeClere 2014 p. 80 (Referencing Cope's Graytree Frog) |
| Northern Redbelly Snake | Ψ (~1) γ (~WodED * WtlED) p(~1) | "This snake is generally considered a woodland species…It often occurs in grassland or along woodland edges near marshes, lakes, or other water sources" LeClere 2014 p. 245 |
|  | Ψ (~1) γ (~WodED * WtrED) p(~1) |  |

Literature Cited

1. LeClere JB. A field guide to the amphibians and reptiles of Iowa. Ecouniverse, Rodeo, New Mexico; 2014.
2. Oldfield B, Moriarty JJ, Breckenridge WJ. Amphibians & reptiles native to Minnesota. University of Minnesota Press, Minneapolis, Minnesota; 1994.
